# Understanding variations in diarrhea management across healthcare facilities in Bangladesh: a formative qualitative study

**DOI:** 10.3855/jidc.17260

**Published:** 2023-05-31

**Authors:** Nour Elshabassi, Stephanie C Garbern, Rochelle K Rosen, Monique Gainey, Sabiha Nasrin, Nur H Alam, Sufia Sultana, Tahmida Hasnin, Adam C Levine

**Affiliations:** 1 School of Public Health, Brown University, Providence, RI, United States; 2 Department of Emergency Medicine, Warren Alpert Medical School, Brown University, Providence, RI, United States; 3 Center for Behavioral and Preventive Medicine, The Miriam Hospital, Providence, RI, United States; 4 Department of Behavioral and Social Sciences, School of Public Health, Brown University, Providence, RI, United States; 5 Rhode Island Hospital, Providence, RI, United States; 6 International Centre for Diarrhoeal Disease Research, Bangladesh (icddr,b), Dhaka, Bangladesh

**Keywords:** diarrhea, qualitative, cholera, Bangladesh, resources, LMICs

## Abstract

**Introduction:**

Acute diarrhea remains a leading cause of morbidity and mortality with over 6.3 billion cases and 1.3 million deaths annually. Despite the existence of standardized guidelines for diarrhea management, wide variability in clinical practice exists, particularly in resource-limited settings. The goal of this study was to qualitatively explore how diarrhea management in Bangladesh varies according to resource availability, clinical setting, and provider roles.

**Methodology:**

This was a secondary analysis of a cross-sectional qualitative study conducted in three diverse hospital settings (district hospital, subdistrict hospital, and specialty diarrhea research hospital) in Bangladesh. A total of eight focus group discussions with nurses and physicians were conducted. Applied thematic analysis was used to identify themes regarding variations in diarrhea management.

**Results:**

Of the 27 focus group participants, 14 were nurses and 13 doctors; 15 worked in a private diarrhea specialty hospital and 12 worked in government district or subdistrict hospitals. Several key themes emerged from the qualitative data analysis: 1) priorities in the clinical assessment of diarrhea 2) use of guidelines versus clinical judgment; 3) variability in clinician roles and between clinical settings influences care delivery; 4) impact of resource availability on diarrhea management; and 5) perceptions of community health workers’ role in diarrhea management.

**Conclusions:**

Findings from this study may aid in informing interventions to improve and standardize diarrhea management in resourceconstrained settings. Resource availability, practices regarding diarrhea assessment and treatment, provider experience, and variability in provider roles are essential considerations when developing clinical tools in low- and middle- income countries.

## Introduction

Acute diarrhea remains a leading cause of morbidity and mortality, particularly in low- and middle-income countries (LMICs), accounting for over 6.3 billion cases and 1.3 million deaths annually [1]. While most cases of diarrhea are self-limiting, more severe cases may require hospitalization for advanced medical management due to complications such as dehydration, malnutrition, and sepsis, which can lead to death if not properly treated [2,3]. The epidemiology of acute diarrhea differs greatly between regions and is caused by a wide variety of pathogens, however, nearly half of all diarrhea hospitalizations in LMICs are due to two key bacteria – *Escherichia coli* (*E. coli*) and *Vibrio cholerae* (*V. cholerae*) [2]. Bangladesh has one of the highest rates of diarrheal disease in the world – with an estimated 450,000 hospitalizations and 4,500 deaths annually due to cholera alone [4]. Cholera, caused by *V. cholerae*, is a potentially fatal bacterial diarrheal disease endemic to Bangladesh with seasonal outbreaks occurring regularly [5]. While Bangladesh has made significant progress in reducing the burden of diarrhea through introducing interventions such as oral rehydration salts (ORS) for dehydration, increasing access to safe water, sanitation and hygiene, and promoting breastfeeding and nutritional education, diarrhea remains one of the main reasons for seeking acute medical care in the country [6–8].

Despite there being standardized guidelines for diarrhea management, including those created by the World Health Organization (WHO) and local healthcare systems, variations in actual diarrhea management practice exist due to human and material resource limitations, epidemiological differences, patient expectations, provider experience, clinical guidelines/tools used, and specialty training in the management of diarrheal disease [9,10]. A 2020 study conducted at government hospitals in Bangladesh found that obstacles to adhering to standardized diarrheal management included difficulties in managing patient treatment expectations (such as desires for intravenous fluids or antibiotic prescriptions), high patient volumes, overcrowding, and unsanitary environmental conditions [10]. Additionally, considerable differences in management practice may exist, particularly between healthcare facility types as well as clinicians.

The provision of healthcare in Bangladesh is carried out by public and private healthcare facilities, including privately-owned hospitals, non-profit organizations, government-run health facilities, and independent clinicians, with potentially large differences in resource availability and consequent care delivery [11]. Since the government offers highly subsidized care for its citizens, publicly funded facilities are most often used by those unable to afford privatized services [11]. Studies have also shown that substantial variations in patient perceptions of hospital service quality exist between private hospitals versus public hospitals in Bangladesh, often leading to patient preference for private facilities despite much higher costs [12–14].

Resource availability across facilities, in combination with a patient’s socioeconomic status (SES), place of residence, or other social determinants of health, could be reasons for varied health outcomes even if strict adherence to guidelines was observed. Government hospitals care for approximately one-quarter of the population living below the national poverty line, and public facilities can become over-extended during seasonal cholera outbreaks when diarrhea cases increase by an estimated 14–24% [15,16]. Research findings also reveal a negative relationship between SES and mortality across age groups, suggesting an additional layer of medical considerations for patients seeking care primarily at public health facilities that could influence the diagnosis to treatment medical paradigm [17,18]. Other influential factors could include a patient’s place of residence [19,20]. A comparative study assessing the prevalence of diarrheal illness in rural versus urban Bangladesh found that individuals living in urban slums were more susceptible to infection [21]. While social factors are a strong predictor of disease etiology, there are other variables to consider, like the availability of clinician expertise, when analyzing the disease to recovery pathway.

Human resources for health (HRH) are often the scarcest commodity with qualified (e.g., physicians, nurses), semi-qualified (e.g., trained community health workers), and unqualified providers (e.g., unlicensed drug vendors) treating patients with diarrhea in Bangladesh [22]. Despite a promising increase in the number of skilled health workers (physicians, nurses, and midwives) in the country, the density of healthcare professionals in Bangladesh remains insufficient with 0.993 skilled health workers per 1,000 people in 2018 – far below the WHO recommended minimum threshold of 4.45 skilled health workers per 1,000 people [23–25]. In absolute terms, this number reflects one of the greatest shortages of HRH globally, with Bangladesh ranking below average compared to other LMICs [26]. Given Bangladesh’s unique and complex clinical context, a nuanced understanding of how environmental, logistical, socio-cultural, and economic factors impact diarrheal management is necessary. Bangladesh presents as a particularly useful case study for exploring why variations in diarrheal management persist in many LMIC contexts, despite the existence of standardized guidelines, and the challenges faced by clinicians, particularly regarding resource availability.

Using data from a qualitative study to develop a new mobile health (mHealth) clinical decision support (CDS) tool for diarrhea management in Bangladesh, this study seeks to examine variations in the attitudes and resources used by clinicians in managing diarrheal disease. Additionally, the study seeks to explore the barriers and enablers to the adoption of new clinical tools from the perspectives of clinicians who care for patients with diarrhea in diverse hospital settings in urban Bangladesh. The findings from this study illustrate how clinical management strategies and perceptions on patient care may vary according to context, resource availability, clinician experience and clinician roles. This improved understanding will allow for better tailoring of new context-appropriate clinical tools and interventions to adequately meet the needs of frontline healthcare workers while considering existing resource limitations.

## Methodology

### Study design

This study is a secondary analysis of qualitative data collected from a series of focus group discussions (FGD) as part of the novel, innovative research for understanding dehydration in adults and kids (NIRUDAK) study. NIRUDAK is an ongoing research effort to develop diagnostic clinical prediction models and incorporate them in a mobile health application (NIRUDAK app) to support clinicians in assessing and treating dehydration in patients with acute diarrhea. FGDs were conducted at three health facilities throughout Bangladesh from November to December 2020 to obtain formative feedback from nurses and physicians about the clinical utility of the NIRUDAK app.27

Ethical approval for the formative phase of the NIRUDAK study was obtained from the International Centre for Diarrhoeal Disease Research, Bangladesh (icddr,b) Research Review Committee and Ethical Review Committee [PR-18077] and the Rhode Island Hospital’s Institutional Review Board [#1244580]. All methods were performed in accordance with relevant guidelines and regulations. Informed consent was obtained in writing from each participant in the native language, Bangla.

Though the research team did not experience challenges with recruiting research participants during the model derivation phase of the NIRUDAK study, barriers arose when recruiting for the FGDs since the timeline overlapped with COVID-19 restrictions. To cope with various regulations imposed, recruitment and data collection occurred virtually. Connectivity issues were experienced that prevented five clinicians from joining the FGDs (1 physician from icddr,b, 2 Narayanganj nurses, 1 Narayanganj physician, and 1 Tongi physician). A total of eight FGDs were hosted across three hospitals: the icddr,b Dhaka Hospital, Narayanganj General Victoria Hospital, and Tongi Sub-district Hospital.

### Study context

Icddr,b is a well-resourced, non-profit and leading health research institute for diarrhea management globally [28]. This specialty hospital has 468 beds and serves over 100,000 patients annually at no cost, the majority of whom present with acute diarrhea. Throughout 2021, icddr,b admitted 92,229 people with an additional 44,683 outpatient visits and 11,709 emergency department visits managed by an experienced clinical staff that includes 89 nurses and 52 physicians. Narayanganj and Tongi are both government-funded hospitals, each about an hour away from the capital city of Dhaka. Narayanganj Hospital, a 100-bed facility, admitted 22,821 patients between January 2021 and December 2021. That same year there were approximately 184,040 outpatient visits and 178,986 emergency department visits managed by a clinical staff of 121 nurses and 29 physicians. Tongi Sub-district Hospital, a 250-bed facility, admitted 14,400 patients during that same year and had 408,000 emergency visits. The clinical staff consisted of 96 nurses and 48 physicians. Both public facilities have small dehydration units that serve patients in the surrounding area. Whereas icddr,b’s bed count is used to exclusively treat patients with diarrhea, beds at Narayanganj and Tongi accommodate patients presenting with diarrhea as well as a variety of other illnesses.

Though key similarities unite all three hospitals located within the administrative division of Dhaka, significant differences also exist. Narayanganj and Tongi hospitals are characterized as being in rural areas, whereas Dhaka is classified as an urban center. According to official Bangladeshi documents, the definition of urban versus rural has varied across censuses. The most recent definition, as of 2011, subdivides urban areas into six distinct groupings, with a population above one million and the existence of city corporations as being key defining features [29]. Rural areas, also referred to as villages, are the smallest administratively recognized territorial designation and are areas with a population less than one million [30]. Tongi Sub-district Hospital and Narayanganj Hospital are located in rural territories one hour away from Dhaka; whereas, icddr,b is located within the capital city.

Consideration of hospital location, whether it is in an urban or rural environment, is important because previous observations show that there are differences in demography and disease distribution between these two settings [19–21]. For example, among the children infected with rotavirus, 34% in Dhaka compared to 6% in a nearby village, exhibited some or severe symptoms of diarrhea [31]. Chronic malnutrition in children under five that results in stunting was found to be 32% in areas without slums and 49% in slum areas [32]. A year later, records showed that the stunting rate in rural areas was 38%, indicating significant variability of malnutrition within urban areas and better nutrition outcomes in rural areas. These variations could indicate differences in the patient populations served by each facility, with icddr,b serving a much wider range of baseline health for patients. Researchers have observed that health outcomes are more greatly impacted by wealth than by geographic location in Bangladesh, with the caveat that poverty is more concentrated in rural areas [33].

Rotavirus, another diarrheal disease, normally peaks in the region between November and January, and overlapped with data collection efforts [2,31]. However, rotavirus primarily affects children < 5 years and therefore it is unlikely that it had a significant impact on the NIRUDAK study since this study enrolled only patients older than 5 years of age with acute diarrhea. There were no other changes in clinical practices or diarrhea interventions co-occurring during the study period other than routine clinical care at icddr,b.

### Data collection and translation

Two FGDs were conducted at each of the government hospitals, one with nurses and one with physicians. Four FGDs were conducted at icddr,b, two with physicians and two with nurses. A pre-written focus group agenda was used to obtain feedback from participants on the development of the NIRUDAK App, a new diarrhea management mHealth tool. Full details of the app’s development process are described elsewhere as the focus of the present study is not on the development of this particular app, but rather on variations in diarrhea management [27,34].

Local research staff led standardized FGDs in Bangla virtually over Zoom, recorded the audio, and transcribed and translated the data. While the number of FGD attendees ranged from two to four individuals, most FGDs were conducted with four participants. This number was kept deliberately low, in accordance with best practices for remote focus groups (Table 1) [35]. All FGDs were facilitated by a member of the Bangladesh-based research team. Additionally, facilitators were part of the transcription and translation team and were supported by a third person. All three individuals were native speakers of the local language (Bangla), from the culture, and understood the clinical and cultural context.

The translation plan included multiple steps. First, the audio data was transcribed into a Bangla transcript, and another member of the team reviewed the audio to ensure correct translation and de-identification. Next, the Bangla transcript was translated into English by a research team member who was proficient in written and spoken English (research team member is also an English teacher). The English transcript was reviewed in Bangladesh by a third team member for accuracy. Finally, a Brown University research team member read each English transcript. Any areas that needed further clarification were identified, in which the Bangladesh-based research team reviewed and resolved all requests for clarification. Once discrepancies were addressed, the English transcript was considered finalized, and the coding process was initiated.

### Analysis

The data analysis plan was informed by applied thematic analysis [36]. Transcripts were coded in the context of this paper’s research question using NVivo qualitative data analysis software. Data was read line by line and systematically categorized into emergent codes that tag barriers to, and perceptions of, care. A codebook was created to index and define each emergent code, and an audit trail was used to document the iterative process of consolidating and creating emergent codes. The coding scheme was finalized with input from a co-investigator, project coordinator, and two analysts. Transcripts were independently coded by two analysts, and discrepancies were resolved to establish inter-coder agreement. Relevant codes were then read in aggregate and summarized for brevity and clarity. Finally, thematic memos interpreting the summaries were written and used in the analysis. The data and memos were interpreted in collaboration with both US- and Bangladesh-based team members for the purpose of extrapolating themes that explored how diarrhea management varies across occupations and the different clinical environments in which the data were collected.

## Results

Twenty-seven clinicians consisting of 14 nurses and 13 doctors participated in a total of eight FGDs across the three hospital sites. Fifteen participants were interviewed at icddr,b and the remaining twelve worked at the government hospitals. Clinical experience ranged from 1–35 years with clinicians at icddr,b having 16.1 years of experience on average compared to 17.5 and 12.8 years at Narayanganj and Tongi respectively. Additional demographic information can be found in Table 2. The comments quoted in this paper represent the voices of 16 participants, none of whom is quoted more than three times. To help preserve the anonymity of participants, we are identifying them only by the type of hospital in which they work.

Five major themes emerged from the qualitative data analysis: 1) priorities in the clinical assessment of diarrhea 2) use of guidelines versus clinical judgment; 3) variability in clinician roles and between clinical settings influences care delivery; 4) impact of resource availability on diarrhea management; 5) perceptions of community health workers’ role in diarrhea management. Each of these themes are explored in depth below.

### Priorities in the clinical assessment of diarrhea

Regarding best practices for clinical assessment, participants reported that their general priorities for assessing a patient with diarrhea usually included: 1) an assessment of dehydration severity; 2) evaluation of suspected etiology of diarrhea; 3) assessment of patient history including relevant co-morbidities and allergies; 4) evaluating for complications such as seizures, electrolyte abnormalities, sepsis, as well as fluid overload from overly aggressive intravenous (IV) fluid administration.

Participants reported that the clinical exam signs they most frequently used to assess severity of dehydration included inability to drink/tolerate oral intake, sunken eyes, delayed skin pinch, decreased radial pulse, lethargy, or irritability. Additionally, participants agreed that evaluating stool appearance, consistency, and frequency was helpful to determine likely diarrhea etiology or severity to assist with decision-making for treatments such as the use of antibiotics.

“So [with] watery stool if [they] have [a] fever… that is one type…with stool having foul smell…that is another type” (subdistrict hospital nurse).

Determining the presence of chronic comorbidities from patient history such as hypertension, cardiovascular disease, and diabetes were noted by participants as important for determining treatment such as fluid resuscitation.

“[For patients with] hypertension [or]…diabetes…then the treatment process will change. And, [one] needs to know in [what] condition is her sugar level, her hypertension level, whether she is taking any medication, and whether the fluid will be prescribed to her or with other fluid for her replacement” (subdistrict hospital nurse).

Additionally, the importance of frequent reassessment and monitoring of patients’ response to fluids was mentioned by several participants, particularly with respect to watching for signs of potential fluid overload in patients with chronic cardiac disease.

“At the initial bolus with half an hour, I will wait…because, you will see when a patient comes with dehydration, her metabolic acidosis, acidic breathing is there, so…when over time hydration is going on… her respiration [rate] will decrease. But [if] it increases suddenly then need to understand…[she may have] fluid overload” (specialty hospital physician).

The presence of fever on patient history or clinical exam was described as important to help determine etiology of diarrhea (particularly viral vs. bacterial infection) and the patient’s treatment plan. However, some felt that fever history was less important, and others felt it was not a priority until after the patient was adequately rehydrated.

“If [a] patient comes, if she has fever with diarrhea…[we] need to ask…is it fever [due to] the diarrhea or [a] food poisoning diarrhea? Or [due to] a medicine [that] this diarrhea happened?” (subdistrict hospital nurse).

Uh… fever history, fever I actually, I do not think that it is a [important] factor because to rehydrate her is the target…fever, to me is less important” (district hospital physician).

Nurses also remarked on the importance of taking a patient’s temperature during the COVID-19 pandemic for appropriately triaging patients and preventing viral spread.

“As we have now… COVID-19 pandemic. So, according [to] that we need to keep in mind…as the patient has temperature now, we will send the patient to the suspected room” (specialty hospital nurse).

Participants agreed that assessment of serious complications such as convulsions and sepsis should be prioritized. For detection of potential sepsis, participants noted that while obtaining blood pressure was often difficult in severely dehydrated patients (due to readings often being too low to measure accurately), checking blood pressure after fluid resuscitation for persistent hypotension as well as assessing for fever were important markers of possible sepsis.

“In case of severe [dehydration] patient… then we will not get her [blood pressure] at all and that time we do not want to check [blood pressure]. Because we know then we are going to get wrong reading. So, when the patient is initially hydrated, then we check [blood pressure]… After initial hydration we may understand, really she is in sepsis or not?” (specialty hospital nurse).

Notably, multiple participants stressed the importance of history of convulsions as a key danger sign due to its association with electrolyte imbalances (particularly due to hypernatremia sometimes due to patient overuse of ORS) and fever (especially in children).

“In diarrhea many people [take] the ORS or bought it from outside. [But] what happens that is many of them have concentrated ORS …[because of that] it has been seen that many of them have hypernatremia convulsion…[and] we give special importance in the case of convulsion having fever….why the convulsion is happening, these causes are very important” (specialty hospital physician).

Opinions on the practicality of obtaining information on a patient’s medication allergies varied, however, clinicians universally recognized it would be valuable information to have. Some clinicians reported that it would take time and effort to collect this information and were doubtful patients would know how to answer properly.

“As a physician, if you want to know our opinion, allergy is important. Yes, universally, we all know it, but according [to] the context of our country, most of the time…a patient cannot say or report it. As in our country that kind of health card or documentary are not kept” (specialty hospital physician).

### Use of guidelines vs. clinical judgment

**Figure 1.**
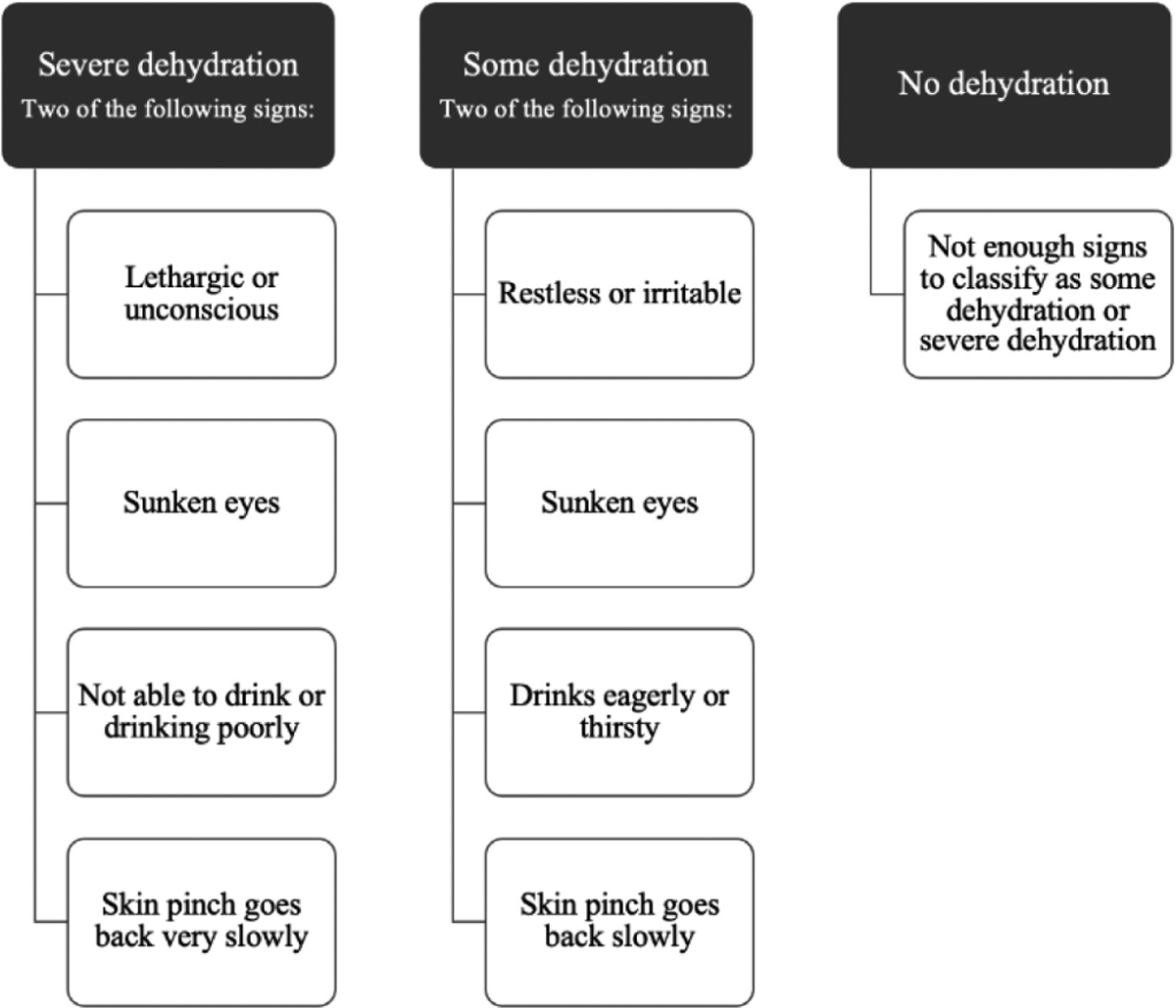
World Health Organization’s Integrated Management of Childhood Illness (IMCI) algorithm for dehydration [30].

Both standardized clinical guidelines and clinical judgement were used by participants in assessing patients with diarrhea. The most common guidelines cited were the Integrated Management of Childhood Illness (IMCI)/Integrated Management of Adult Illness (IMAI) algorithms established via consensus among medical professionals and developed by the WHO for quickly classifying dehydration severity in patients in low-resourced settings; the algorithm is shown in (Figure 1) [37,38]. Clinicians at icddr,b frequently reported using the Dhaka method (Table 3), which was developed at icddr,b, for assessing dehydration severity [39,40]. Icddr,b clinicians referenced using the Dhaka method and treatment guidelines more frequently, and noted they use the Dhaka method for diagnosis and WHO guideline for treatment (i.e. WHO Treatment Plan A, B, C). Government clinicians referenced awareness of icddr,b protocols and trainings but did not specifically refer to using the Dhaka method and instead used WHO guidelines for treatment.

“At icddr,b… we use [the] Dhaka method for classifying [a] patient’s dehydration…when we manage that, then we use WHO that has…the [treatment] recommendation.” (specialty hospital physician)

According to physicians, patient assessment and treatment are often performed using their clinical judgment – a combination of medical knowledge, experience, and taking clinical assessment and patient history. Notably, physicians (rather than nurses) were more critical of decision support tools and clinical guidelines, noting that these resources cannot replace clinician expertise when managing a patient’s diarrhea.

“Normally we [follow our] MBBS curriculum those we have read, plus during internship [what] we learnt, mainly [use] that…[as a] guideline. Now, we do not read [the] guideline regularly. So, based on…our acquired knowledge and our clinical practice, clinical knowledge, based on…physical appearance, history, clinical examination, general condition all these we consider, after that we give treatment.” (subdistrict hospital physician)

Additionally, participants also acknowledged that despite the use of guidelines, the assessment of diarrhea may vary between clinicians. Particularly regarding assessment of dehydration, there was some degree of subjectivity in categorizing dehydration severity.

“When we do assessment, it will differ…man to man it varies…[their] opinions will differ; [what] I will think severe; to him/her it might not be.” (specialty hospital nurse)

Lastly, it was felt that clinician experience plays an important role in the recognition and treatment of dehydration severity, therefore participants felt a clinical support tool would be particularly helpful for new physicians in training or less experienced clinicians since they will be given a treatment plan to consider based on the patient’s symptoms.

“Many times [the facility will] have new nurses, many times [it will] have new doctors. Then, by seeing these [output screens] to understand [management]…will be helpful.” (specialty hospital nurse).

### Variability in clinician roles and between clinical settings influences care delivery

All participants reported some clear distinctions between physician and nurse roles. For example, only physicians could prescribe medications such as antibiotics, while nurses were primarily responsible for administering medicine, monitoring patient progress, and keeping the physician abreast as needed. However, variability in the treatment of severely dehydrated patients arose when discussing clinician responsibilities across contexts. According to nurses and physicians at icddr,b, nurses could independently decide to give a patient an IV or ORS based on their assessment of dehydration severity. Conversely, nurses at government hospitals were usually described as being limited to carrying out physician orders.

“Yes, sister our patient actually come to the emergency, take admission over there. From there, doctor gives order in the treatment sheet that the patient need this much liter fluid…they give order and we work according [to] that order” (district hospital nurse).

Hospitals varied regarding who could complete a patient intake assessment. At icddr,b, intake was the responsibility of the on-duty nurse, but at government hospitals, participants reported that intern doctors or emergency physicians have this responsibility. Additionally, at icddr,b, participants reported that nurses could diagnose dehydration severity, although only physicians could diagnose sepsis and prescribe antibiotics.

“Suppose have hundred patients, in that case, nurses will diagnose fifty patients, doctors will diagnose fifty patients” (specialty hospital nurse).

“If I have to diagnosis sepsis, then in that case, you need a doctor. Even sisters [nurses] cannot do it, to be honest to diagnose sepsis. However, if it is dehydration, doctor- nurse both can do…community health workers cannot do” (specialty hospital physician).

Conversely, at government hospitals physicians were reportedly solely responsible for assessing the patient’s dehydration severity and directing nurses on how to proceed with the patient’s treatment plan.

“After that, here is a practice that doctors give order, according [to] her diarrheal dehydration status they will detect that she [the patient] will be given this much liter fluid replacement” (subdistrict hospital nurse).

A nurse working at a government hospital explained that if a patient was severely dehydrated and had an electrolyte imbalance, they sometimes used their clinical judgement to increase the amount of saline given. Although nurses were not diagnosing patients, they used their clinical knowledge and experience to modify treatments, and subsequently keep physicians informed of cases that were not adequately responding to treatment.

“If we see that in case of dehydration electrolyte imbalanced is more, in that case, sometimes we, of our own [judgement], increase saline. After that, many times [we] call the doctor to increase drops that has severe dehydration [persist]” (subdistrict hospital nurse).

Lastly, clinicians at government hospitals were more likely to recommend additional health interventions like the prescription of an anti-emetic or probiotic. At icddr,b treatment plans were restricted to administering IVs, antibiotics, and antipyretic medication. For example, when provided with a discussion case scenario of a severely dehydrated patient, a government physician responded with the following statement:

“After hospitalization…obviously she needs to have fluid. In that case, cholera saline that we usually used…with it she needs to have anti-emetic. As antibiotic, we can use macrolides group and with it is obvious to have in oral, oral dehydration saline and with it we can add probiotic” (subdistrict hospital physician).

### Impact of resource availability on diarrhea management

Nurses and physicians at all healthcare facility types reported how demands on clinician time during periods of high patient volume often led to the prioritization of initiating treatment over performing patient assessments. For instance, during epidemic periods (i.e., cholera season), participants described how high patient caseloads limited one’s ability to perform any additional (even minor) tasks such as taking additional vital signs or other measurements and conducting thorough patient assessments.

“In emergency, sister, many times it is not obvious. [We can have] a diarrhea patient comes, [then] in a few minutes a crashed patient arrives. I will manage this or that?! A cut patient, torn patient, hand is broken, leg is broken or many times patient comes having poison. So, I have to check this very quickly.” (subdistrict hospital physician)

Specifically, clinicians at icddr,b reported that the scarce time available for each patient made it difficult to prioritize accurately assessing for comorbidities or other complications versus implementing treatments such as rehydration immediately. Instead, doctors and nurses reported that one reason they preferred providing immediate treatment rather than diagnosis was because the time it takes to diagnose a patient could be used to treat another severely dehydrated patient. When asked about the importance of assessing a patient’s blood pressure (BP) as part of a routine clinical assessment, an icddr,b nurse responded with the following statement:

“To manage a patient, I might hamper another patient. It is [a] waste of time, I think.” (specialty hospital nurse)

Other reasons that icddr,b clinicians reported for prioritizing treatment over assessment include concern for a patient’s worsening condition due to comorbidities and the fact that taking measurements of a severely dehydrated and unconscious patient is not an efficient use of time and presents challenges to the healthcare staff working at that time.

“Another thing is that in severe dehydration, many patients come who are almost unconscious, so making the patient stand to take [their] weight or making him sit to take weight… for an elderly patient it will be very tough” (specialty hospital physician).

Physicians and nurses reported that collecting information, like BP, may be helpful, though dependent on resource availability, as not all clinical contexts have equipment for measuring BP. This was particularly relevant regarding taking children’s BP as many facilities do not have pediatric sized BP cuffs.

“Actually, we have adult [blood pressure] cuff…this type [child blood pressure cuff]…[we] do not have, it is not available here. So, I do not think that for 7–8-year-old child’s [blood] pressure can be measured in our government hospital” (subdistrict hospital physician).

Some physicians and nurses found BP especially helpful for determining how much saline to give a patient, however others expressed that BP is non-detectable if a patient is severely dehydrated and therefore unnecessary data to collect.

“In severe dehydration case BP diastolic, systolic BP, mean we may get or not [in severe dehydration usually BP is not recordable]; this is normal. For this I think that…[BP] is not needed” (specialty hospital nurse).

### Perceptions of community health workers’ role in diarrhea management

Population density and the density of skilled health workers is much greater in cities than in rural Bangladesh [41]. Community health workers (CHWs) help fill this gap and provide essential healthcare delivery services, especially in rural Bangladesh. Clinicians described their opinions regarding the role of CHWs in diarrhea management and their use of CDS tools to standardize diarrhea management. Physicians expressed concern that nurses, CHWs, and those working in remote areas might not have the clinical training, knowledge, and experience to properly conduct clinical assessments, take correct medical decisions, and record information like a patient’s pulse or BP, thereby resulting in an incorrect diagnosis. When asked by the interviewer about having CHWs use a decision support tool in remote areas, this participant responded with the following:

“It is said that many times they do not understand [what] decreased pulse [is] because …they do not learn it” (specialty hospital physician).

Participants expressed concern with CHWs not having sufficient medical knowledge to recognize patient symptoms accurately enough to properly diagnose. Though this served as a barrier, participants reported that CHWs can get trained in assessing clinical signs of dehydration and recognize when specialized care is needed. Most clinicians felt strongly that CHWs do not have the clinical training to properly administer recommended treatments. Consequently, participants reportedly felt that a decision support tool should only be used by CHWs as a referral tool (i.e., for referral to another health facility or hospital) rather than as a treatment tool.

“A community health worker…assesses a patient at the house, maybe the child’s relatives are not aware [how] dehydrated the patient is, he assesses that severe dehydration is there and she needs to send to the center, either to any hospital. You can send him to local hospitals too…then it will be a great help” (specialty hospital physician).

Many participants reportedly felt that a decision support tool that is used for referring patients to the hospital would be especially useful in rural parts of the country. Both types of clinicians expressed, however, that the application should mostly be used by nurses and physicians since they can utilize their medical training and rely on their clinical judgement.

## Discussion

While there are standardized guidelines for diagnosing and treating diarrhea, variability in diarrhea management exists across clinical contexts, experience, and role, and are exacerbated by resource limitations. Time constraints, a lack of clinical training and experience, and a disproportionate ratio of clinicians to patients, particularly during cholera epidemics, shapes the environment in which diagnosis and treatment are provided. The interplay between social factors and medical conditions also plays a role in the patient’s existing health and subsequent health outcomes due to the quality of care and facilities they have access to.

Findings from this study also reaffirm previous studies that have documented human resource issues in the management of diarrhea [9,10,22,41]. These include the tension between difficulties in adhering to standardized guidelines and providing clinical care in resource limited settings [10]. A recent ethnographic study in Bangladesh comprising over 130 informal interviews and systematic clinical ethnographic observation found that physicians took a limited medical history, frequently determined dehydration status visually rather than via physical examination, and provided IV fluids in 90% of observed encounters, even when patients were not severely dehydrated [10]. The team suggested that physicians prescribed treatment plans that were partially motivated by patient expectations of antibiotic prescription or IV treatment instead of ORS, or due to conflicts of interest, such as when physicians refer clients to their private practice [10]. The present study has similarly identified an additional motivational factor, which includes a preference to prioritize rapid treatment over more thorough assessments to guide treatment, in order to increase the number of patients treated during times of high patient volume and extreme resource constraints.

Additionally, this study found that differences in the type of guidelines used across facilities and variable access to medical equipment needed to assess patient vitals leads to inconsistent diarrheal management. This is further influenced by variations in clinician experience and role as demonstrated by the greater autonomy of icddr,b nurses when diagnosing and treating dehydrated patients. Conversely, the role of nurses at government hospitals appears to be more strictly limited to carrying out physician orders, although the exact role and expectations were less clearly defined. For example, nurses working at government hospitals report intervening in a patient’s treatment plan if they felt it was necessary and subsequently informing physicians of the changes made. This suggests that nurses may have different degrees of autonomy based on circumstances such as resource limitations resulting in fewer physicians able to direct orders. Tensions between providing adequate health care in a context with greater resource and time constraints might force nurses to take on tasks that directly conflict with their role. Given the fluidity of nursing responsibilities based on clinical context, future interventions to improve diarrhea management must account for a range of nurse-specific needs and roles to ensure optimal patient health outcomes. Furthermore, multiple studies, have demonstrated how mHealth and CDS tools improve adherence to clinical guidelines, suggesting that access to these resources can play a role in reducing variability in disease management [42,43].

Given icddr,b’s designation as a research institute specializing in diarrhea, clinician care primarily focuses on evidence-based management strategies; whereas government clinicians may be highly dependent on patient satisfaction and meeting expectations for treatments that address symptoms as well as demands for antibiotics and other prescription drugs. This may explain why clinicians at government hospitals prescribe patients a wider variety of medications than clinicians at icddr,b.

Use of a dehydration CDS tool by CHWs as a diagnostic or treatment aid was discouraged by nurses and physicians at all study sites due to a perceived lack of clinical knowledge that could result in improper use. Instead, clinicians suggested that CHWs receive training to recognize dehydration symptoms so they can input the information in the application and refer patients to the hospital as necessary. According to the participants, an mHealth application would also be especially helpful as a training tool for new clinicians. These findings suggest that a medical decision support tool should be developed with the realization that its utility will vary according to the experience and training of the user.

### Limitations

Data analyzed for this study was collected in the context of trying to develop a CDS tool for assessing dehydration severity in diarrheal disease. Therefore, information in the FGDs mostly discussed assessments, made few references to treatment, and did not directly ask questions related to resource availability and therefore may not be representative of all clinician perspectives on the topic. Additionally, as this study was conducted in urban hospitals in Dhaka, the findings may not be generalized to outpatients, rural populations, or other countries with different resource availability and medical education systems. Attitudes of other types of healthcare providers, such as CHWs or pharmacists, were not captured but would add valuable insights because they are the primary source of care in rural settings. Future studies can address this limitation by gathering information from different clinical contexts like outpatient clinics or pharmacies. Finally, a wider geographic location which includes insights from clinicians providing care at both rural and urban environments would be important to explore further.

## Conclusions

Novel interventions aimed at improving the care of patients with acute diarrhea in resource-limited settings must consider how clinical management of diarrhea is greatly influenced by variations in resource availability, prioritization of clinical assessments, clinician role, and clinical context. Despite standardized guidelines for diarrhea management, individual clinicians’ practice styles vary based on experience, resource availability, and their priorities for patient management. Respecting clinician autonomy and expertise to make decisions, particularly for atypical case presentations are crucial factors to consider when creating CDS tools. As both material and human resource limitations may influence the prioritization of treatment versus assessment, the introduction of diagnostic tools should emphasize the improved patient outcomes and resources saved through more accurate diagnoses. Variations in nurse roles and responsibilities, and the potential use of CDS tools to aid in referral decisions by CHWs suggest additional opportunities for tool customization. Future research aimed at exploring clinician perspectives for improving diarrhea management using CDS tools, and how diarrhea management varies in other clinical contexts such as outpatient settings or by other clinician types are warranted.

## Figures and Tables

**Table 1.** Breakdown of focus group discussions (FGD).

FGD	Hospital	Hospital type	Clinician role	Number of participants
1	icddr,b	Sub-specialty hospital	Physician	4
2	icddr,b	Sub-specialty hospital	Physician	3
3	icddr,b	Sub-specialty hospital	Nurse	4
4	icddr,b	Sub-specialty hospital	Nurse	4
5	Narayanganj	District hospital	Nurse	2
6	Narayanganj	District hospital	Physician	4
7	Tongi	Sub-District hospital	Nurse	4
8	Tongi	Sub-District hospital	Physician	3

icddr,b: International Centre for Diarrhoeal Disease Research, Bangladesh.

**Table 2.** Demographics of participants in the focus group discussions.

Characteristics	n (%)
**Age (years)**	
25–34	6 (22.2)
35–44	16 (59.3)
45–54	4 (14.8)
55–64	1 (3.7)
**Gender**	
Male	9 (33.3)
Female	18 (66.7)
**Position and degree**	
Nurse	14 (51.9)
Diploma	5 (35.7)
Bachelor’s degree	4 (28.6)
Master’s degree	5 (35.7)
Physician	13 (48.1)
Bachelor of Medicine, Bachelor of Surgery	8 (61.5)
Master’s degree	5 (38.5)
**Monthly household income in Taka**	
10,001–50,000	7 (25.9)
50,001–100,000	6 (22.2)
100,000+	14 (51.9)
Experience in current position in years, mean (SD)	10.3 (9.0)
**Hospital Location**	
icddr,b	15 (55.6)
Tongi Upazilla or Sub-District Hospital	7 (25.9)
Narayanganj General Victoria District Hospital	5 (18.5)

a icddr,b: International Centre for Diarrhoeal Disease Research, Bangladesh.

**Table 3.** Dhaka method of assessing dehydration [34].

Parameters		Clinical findings	
Condition*	Normal	Irritable/less active*	Lethargic/comatose
Eyes	Normal	Sunken	
Mucosa	Normal	Dry	
Thirst*	Normal	Thirsty*	Inability to drink*
Skin turgor*	Normal	Reduced*	
Radial pulse*	Normal		Uncountable/absent*
Diagnosis	No dehydration	If at least two signs, including one key sign are present, diagnose some dehydration	If some dehydration plus one of the above key signs are present, diagnose severe dehydration
Estimated bodyweight loss	0–4%	5–10%	>10%

* Key sign.
